# An FGF2-Derived Short Peptide Attenuates Bleomycin-Induced Pulmonary Fibrosis by Inhibiting Collagen Deposition and Epithelial–Mesenchymal Transition via the FGFR/MAPK Signaling Pathway

**DOI:** 10.3390/ijms26020517

**Published:** 2025-01-09

**Authors:** Mengwei Wang, Yuanmeng Sun, Yanzhi Zhao, Xinyi Jiang, Teng Wang, Junye Xie, Xiuling Yu, Shujun Guo, Yibo Zhang, Xiaojia Chen, An Hong

**Affiliations:** 1 Institute of Biomedicine & Department of Cell Biology, College of Life Science and Technology, Jinan University, Guangzhou 510632, China; mengweiii@163.com (M.W.); sunyuanmeng111@163.com (Y.S.); 17789734623@163.com (Y.Z.); j838073220@163.com (X.J.); tengwang@stu2022.jnu.edu.cn (T.W.); xiejunyep@163.com (J.X.); 15332051603@163.com (X.Y.); gsj2011@jnu.edu.cn (S.G.); zyb_87@hotmail.com (Y.Z.); 2 State Key Laboratory of Bioactive Molecules and Druggability Assessment, Jinan University, Guangzhou 510632, China; 3 National Engineering Research Center of Genetic Medicine, Guangzhou 510632, China; 4 Guangdong Province Key Laboratory of Bioengineering Medicine, Guangzhou 510632, China; 5 Guangdong Provincial Biotechnology Drug & Engineering Technology Research Center, Guangzhou 510632, China; 6 MOE Key Laboratory of Tumor Molecular Biology, Jinan University, Guangzhou 510632, China

**Keywords:** pulmonary fibrosis, FGF2-derived short peptide, FGFR/MAPK, epithelial–mesenchymal transition (EMT), collagen deposition

## Abstract

Following the COVID-19 pandemic, the prevalence of pulmonary fibrosis has increased significantly, placing patients at higher risk and presenting new therapeutic challenges. Current anti-fibrotic drugs, such as Nintedanib, can slow the decline in lung function, but their severe side effects highlight the urgent need for safer and more targeted alternatives. This study explores the anti-fibrotic potential and underlying mechanisms of an endogenous peptide (P5) derived from fibroblast growth factor 2 (FGF2), developed by our research team. Using a bleomycin-induced pulmonary fibrosis mouse model, we observed that P5 alleviated fibrosis by inhibiting collagen deposition, as confirmed by CT scans and histological staining. In TGF-β-induced cell models, P5 effectively suppressed collagen deposition and epithelial–mesenchymal transition (EMT). Transcriptome analysis highlighted pathways related to receptor binding, extracellular matrix organization, and cell adhesion, with KEGG analysis confirming FGFR/MAPK signaling inhibition as the primary mechanism underlying its anti-fibrotic effects. In summary, our study demonstrates that P5 significantly attenuates pulmonary fibrosis through the inhibition of EMT, collagen deposition, and FGFR/MAPK signaling, providing a promising therapeutic approach for fibrosis.

## 1. Introduction

Pulmonary fibrosis is a chronic, progressive interstitial lung disease (ILD) characterized by alveolar damage, fibroblast activation, inflammation, and the formation of scar tissue [1]. The causes of pulmonary fibrosis are multifactorial, encompassing various factors such as occupational exposures (e.g., dust, coal) [2,3,4], autoimmune diseases (e.g., rheumatoid arthritis) [5], medications (e.g., amiodarone) [6], radiation exposure [7], and genetic mutations [8,9,10]. Notably, idiopathic pulmonary fibrosis (IPF) is defined by its unknown etiology, making it the most common form of pulmonary fibrosis. The disease primarily affects individuals over the age of 50, with a higher prevalence in men [11,12]. Recent studies have highlighted a global rise in IPF prevalence, accompanied by a poor prognosis. The median survival time following diagnosis is only 3–5 years, and the mortality rate of IPF is higher than that of several cancers [13,14]. These concerning trends emphasize the urgent need for enhanced understanding and more effective treatment options for IPF.

The pathogenesis of pulmonary fibrosis involves fibroblast and myofibroblast proliferation and excessive extracellular matrix (ECM) deposition [15]. The FGF (fibroblast growth factor) family plays a significant role in tissue repair and fibroblast proliferation, suggesting its importance in pulmonary fibrosis [16]. FGF2 is overexpressed in epithelial, endothelial, and smooth muscle cells in IPF patients, promoting fibroblast proliferation [17]. FGF1, which binds to FGFRs, shows both anti-fibrotic effects, such as inhibiting myofibroblast differentiation and promoting epithelial cell proliferation, and pro-fibrotic effects by activating the MAPK pathway [18,19,20]. FGF7 and FGF10 help protect against fibrosis by promoting epithelial regeneration [21,22,23,24]. FGF9 and FGF18 support fibroblast survival and inhibit fibroblast-to-myofibroblast differentiation [25]. FGF21 may reduce fibrosis by activating the Nrf2 pathway, suggesting its potential as a therapeutic agent [26]. FGFRs (fibroblast growth factor receptors) are upregulated in IPF and appear to regulate downstream signaling pathways like ERK and Smad, which may have anti-fibrotic effects [27,28,29]. The soluble mutant msFGFR2 has been shown to inhibit TGF-β1-induced fibroblast proliferation and reduce fibrosis, presenting it as a potential therapeutic candidate [30]. However, the precise contribution of endogenous FGFR signaling in IPF remains unclear, and further studies are needed to explore the role of angiogenesis in IPF.

In our previous research, we demonstrated that P5 inhibits FGFR2 and androgen receptor (AR) signaling, suggesting its potential as an acne antagonist [31]. Moreover, we discovered through Isothermal Titration Calorimetry (ITC) experiments that the truncated peptide P5 from FGF2 interacts with tyrosine kinase family members, including FGFR1-FGFR4, Vascular Endothelial Growth Factor Receptor 2 (VEGFR2), Human Epidermal Growth Factor Receptor 2 (HER2), Platelet-Derived Growth Factor Receptor Alpha (PDGFRα), Platelet-Derived Growth Factor Receptor Beta (PDGFRβ), Insulin-Like Growth Factor 1 Receptor (IGF1R), and Hepatocyte Growth Factor Receptor (HGFR) [32]. Molecular docking simulations first established a binding model of the bFGF ligand with the extracellular D2-D3 region of FGFR2 (Figure S1A), showing that bFGF (yellow) stably binds to the D2 and D3 domains of FGFR2 (blue). Based on this model, the P5 (green) was found to also bind stably to the D2-D3 region of FGFR2 (Figure S1B) through multiple non-covalent interactions, including hydrogen bonds, hydrophobic forces, and salt bridges (Figure S1C). The binding site of P5 overlaps with that of bFGF, suggesting that P5 may cause steric hindrance to bFGF binding, potentially inhibiting FGFR2-mediated signaling and acting as a competitive inhibitor. Given that excessive FGFR signaling activation is closely linked to pulmonary fibrosis, inhibiting this overactivation is crucial. However, current FGFR inhibitors are limited [33,34]. Although Nintedanib, which targets FGFR1/2/3, VEGFR1/2/3, and PDGFRα/β tyrosine kinase domains, has been developed, it has drawbacks such as resistance, significant side effects, and a narrow range of indications, limiting its effectiveness in treating pulmonary fibrosis [35,36].

Our study found that a naturally degraded fragment of FGF2, named P5, can bind to FGFRs and inhibit their activation. This study aims to explore the effects and regulatory mechanisms of P5 on pulmonary fibrosis in the context of a bleomycin-induced pulmonary fibrosis mouse model, providing insights into the disease’s pathogenesis and potentially offering a new therapeutic strategy.

## 2. Results

### 2.1. Therapeutic Effects of P5 on BLM-Induced Pulmonary Fibrosis in Mice

**Figure 1:**
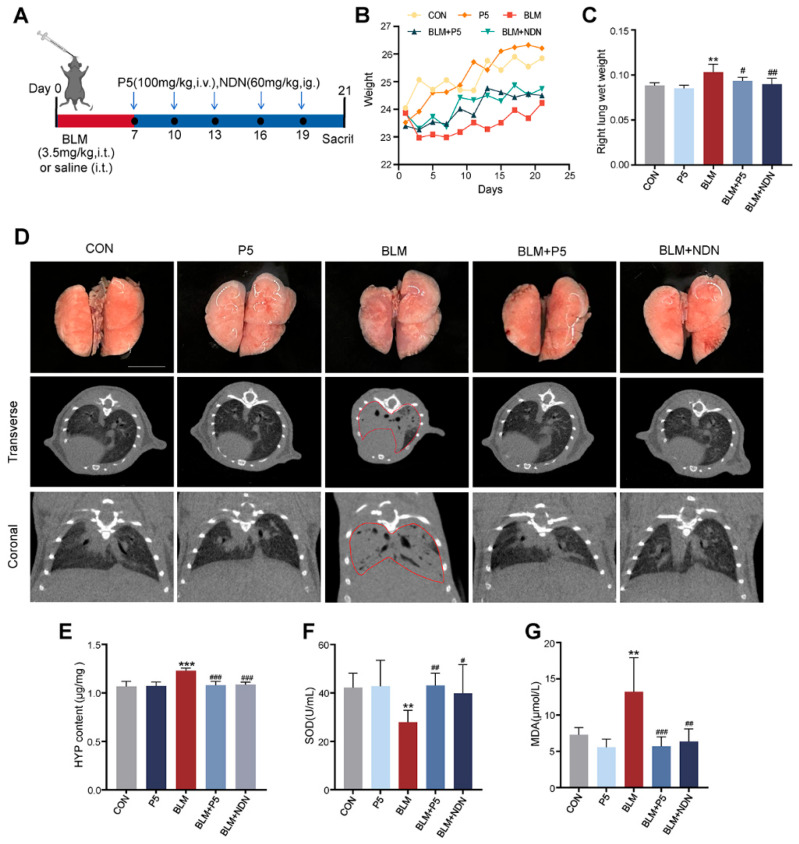
The effect of derivative P5 on bleomycin-induced pulmonary fibrosis in a mouse model. (**A**) Experimental design overview. Mice were administered a single dose of 3.5 mg/kg bleomycin (BLM) on day 1 to induce pulmonary fibrosis. Treatments with 100 mg/kg P5 or 60 mg/kg Nintedanib were started on day 7 and given every three days, with final analysis on day 21. (**B**) Changes in mouse body weight during the experiment. (**C**) Ratio of right lung wet weight. (**D**) Representative images of lung tissue and CT scans. (**E**) Hydroxyproline (HYP) content in lung tissue. (**F**) Superoxide dismutase (SOD) activity in serum. (**G**) Malondialdehyde (MDA) content in serum. Data are presented as mean ± SD (*n* = 3–6). For comparisons among multiple groups, a one-way analysis of variance (ANOVA) was applied. ** *p* < 0.01, *** *p* < 0.001 compared to the control group (CON); # *p* < 0.05, ## *p* < 0.01, ### *p* < 0.001 compared to the BLM group. CON: control; BLM: Bleomycin; NDN: Nintedanib. Scale bars = 5 mm in B.

To evaluate the therapeutic effects of the P5 on bleomycin (BLM)-induced pulmonary fibrosis, mice in the experimental group were administered BLM (3.5 mg/kg) via intratracheal injection to induce lung fibrosis. Starting on day 7, the mice were treated with either P5 or Nintedanib and were sacrificed on day 21 for further analysis (Figure 1A). P5 and Nintedanib mitigated BLM-induced body weight loss (Figure 1B) and reduced the wet weight of the right lung (Figure 1C). Macroscopic examination and CT scans of lung tissues revealed extensive fibrotic lesions with uneven density and structural disorganization in BLM-treated mice, which were notably improved by P5 and Nintedanib treatment, restoring the lung tissue to a more normal appearance (Figure 1D). Hydroxyproline (HYP) content analysis showed a significant increase in HYP levels following BLM treatment, which was substantially reduced by P5 and Nintedanib, indicating an inhibition of lung fibrosis (Figure 1E). Furthermore, P5 and Nintedanib effectively restored SOD activity and reduced MDA levels (Figure 1F,G). These findings suggest that P5 can effectively alleviate BLM-induced pulmonary fibrosis. Notably, the optimal concentration of P5 for this effect was determined to be 100 mg/kg, a dosage that does not cause organ damage (Figure S2).

### 2.2. P5 Ameliorates BLM-Induced Lung Tissue Damage in Mice

**Figure 2:**
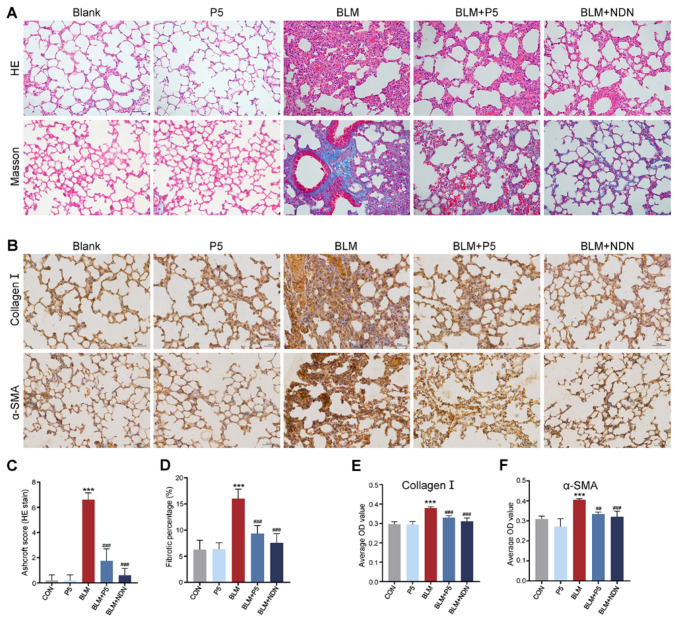
Histological and immunohistochemical analysis of the effect of P5 on bleomycin-induced pulmonary fibrosis. (**A**) Representative images of HE and Masson staining of lung tissue. (**B**) Representative images of immunohistochemical staining for collagen I and α-SMA. (**C**) Quantitative analysis of HE staining scores. (**D**) Percentage of fibrotic area in Masson staining. (**E**) Average optical density (OD) value of Collagen I. (**F**) Average OD value of α-SMA. Data are presented as mean ± SD (*n* = 4–8). For comparisons among multiple groups, a one-way analysis of variance (ANOVA) was applied. *** *p* < 0.001 compared to the control group (CON); ## *p* < 0.01, ### *p* < 0.001 compared to the BLM group. Scale bars = 50 µm in A, 50 µm in B.

To further evaluate the effects of the P5 on bleomycin (BLM)-induced pulmonary fibrosis, we performed histological and immunohistochemical analyses of lung tissue. Histological analysis showed that BLM caused severe alveolar damage and fibrosis, as evidenced by HE and Masson staining. P5 and Nintedanib treatments significantly reduced alveolar destruction and collagen deposition, alleviating fibrosis (Figure 2A). Immunohistochemical staining demonstrated that BLM treatment significantly increased the expression of collagen I and α-SMA; however, P5 and Nintedanib treatments markedly reduced the levels of these markers (Figure 2B–F). In summary, P5 effectively mitigates BLM-induced pulmonary fibrosis by reducing collagen deposition in lung tissue.

### 2.3. P5 Reduces α-SMA and Collagen I in TGF-β1-Activated Cells

**Figure 3:**
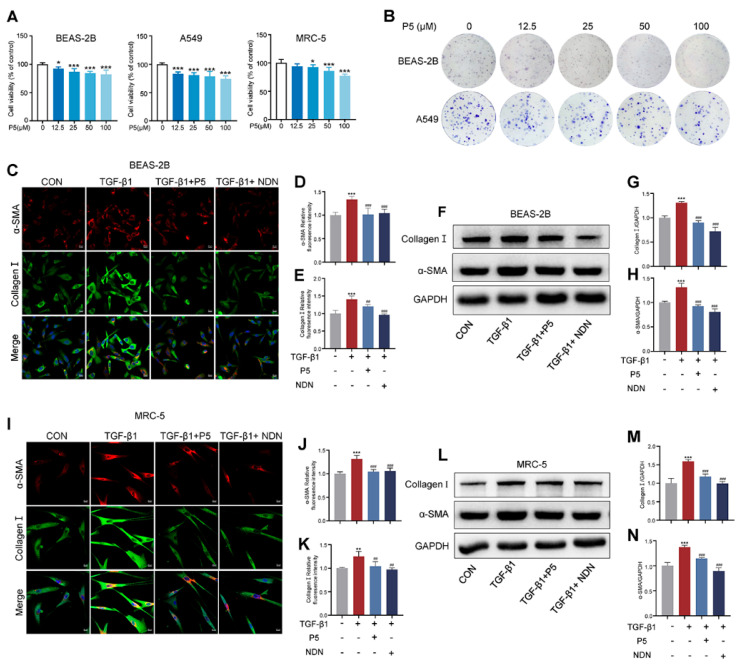
P5 attenuates TGF-β1-induced fibrotic changes in BEAS-2B, A549, and MRC-5 cells. (**A**) Cell viability of BEAS-2B, A549, and MRC-5 cells treated with various concentrations of P5 (0, 12.5, 25, 50, 100 μM) for 24 h. (**B**) Colony formation assay of BEAS-2B and A549 cells treated with different concentrations of P5 (0, 12.5, 25, 50, 100 μM). (**C**) Immunofluorescence staining of α-SMA (red) and collagen I (green) in BEAS-2B cells treated with TGF-β1 (10 ng/mL) and/or P5 (100 μM) or Nintedanib (NDN, 1 μM) for 24 h. (**D**,**E**) Quantitative analysis of immunofluorescence intensity for α-SMA and Collagen I in BEAS-2B cells. (**F**) Western blot analysis of collagen I and α-SMA expression in BEAS-2B cells. (**G**,**H**) Quantification of collagen I/GAPDH ratio and α-SMA/GAPDH ratio in BEAS-2B cells. (**I**): Immunofluorescence staining of α-SMA (red) and collagen I (green) in MRC-5 cells treated with TGF-β1 (10 ng/mL) and/or P5 (100 μM) or Nintedanib (NDN, 1 μM) for 24 h. (**J**,**K**) Quantification of collagen I/GAPDH ratio and α-SMA/GAPDH ratio in MRC-5 cells. (**L**) Western blot analysis of collagen I and α-SMA expression in MRC-5 cells. (**M**,**N**) Densitometric analysis of Western blot results for collagen I and α-SMA in MRC-5 cells. Data are presented as mean ± SD (*n* = 3–4). For comparisons among multiple groups, a one-way analysis of variance (ANOVA) was applied. * *p* < 0.05, ** *p* < 0.01, *** *p* < 0.001 compared to the control group (CON); ## *p* < 0.01, ### *p* < 0.001 compared to the TGF-β1 group. The ‘+’ symbol represents ‘presence’ and the ‘–’ symbol represents ‘absence’. Scale bars = 20 µm in C, 20 µm in (**I**).

Activated fibroblasts are the primary source of collagen deposition, with TGF-β1 being the most common cytokine driving fibroblast activation [37,38]. To evaluate the effect of P5 on TGF-β1-induced fibroblast activation, we conducted a series of experiments. P5 demonstrated a dose-dependent inhibition of cell proliferation and activation in BEAS-2B, A549, and MRC-5 cells, as shown by CCK-8 and colony formation assays (Figure 3A,B and Figure S3A,B). Western blot analysis of TGF-β1-treated BEAS-2B cells revealed a significant dose-dependent reduction in fibrotic markers collagen I and α-SMA, with 100 µM identified as the optimal intervention concentration (Figure S3C–E). Immunofluorescence assays further confirmed that P5 markedly reduced the expression of α-SMA and collagen I in both BEAS-2B and MRC-5 cells after TGF-β1 induction (Figure 3C–E,I–K). Further confirmation by Western blot analysis showed that P5 treatment significantly reduced TGF-β1-induced protein expression of α-SMA and collagen I (Figure 3F–H,L,M, and Figure S3F–H).

### 2.4. Transcriptomic Analysis of TGF-β1-Induced BEAS-2B Cells

**Figure 4:**
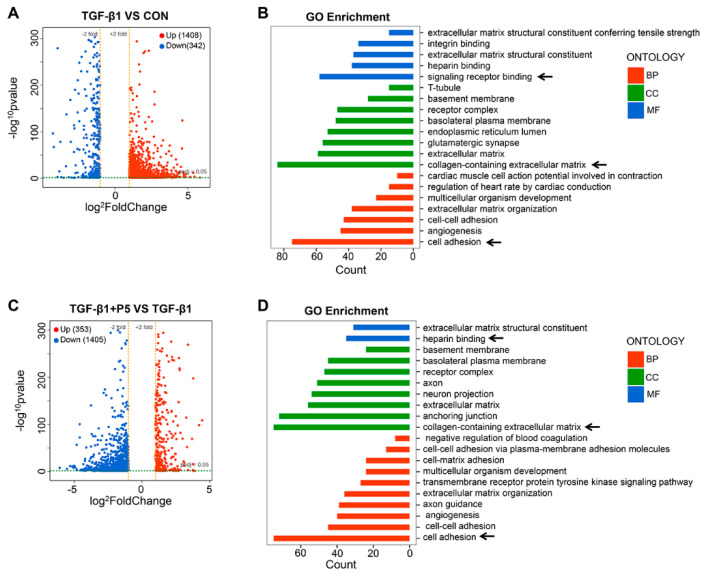
Effect of P5 on TGF-β1-induced gene expression. (**A**) Volcano plot analysis of differentially expressed genes (DEGs) between the TGF-β1-treated group and the control group (CON). (**B**) GO enrichment analysis between the TGF-β1-treated group and the control group (CON). The black arrows indicated the main pathways affected by TGF-β1. (**C**) Volcano plot analysis of DEGs between the TGF-β1 + P5 group and the TGF-β1 group. (**D**) GO enrichment analysis between the TGF-β1 + P5 group and the TGF-β1 group. The black arrows indicated the main pathways affected by P5. BP: Biological processes; MF: Molecular functions; CC: Cellular components. Arrows indicate significantly enriched terms.

To further investigate the impact of the derivative peptide P5 on gene expression changes induced by TGF-β1, we performed RNA sequencing and analyzed differential expression genes (DEGs) using volcano plots and gene ontology (GO) enrichment. The DEG volcano plot revealed 408 upregulated genes and 342 downregulated genes between the TGF-β1 and control (CON) groups (Figure 4A). GO enrichment analysis indicated that these DEGs were primarily involved in extracellular matrix (ECM) organization, integrin binding, signal receptor binding, and cell adhesion, suggesting that TGF-β1 promotes ECM remodeling and fibrosis through these pathways (Figure 4B). In comparing DEGs between the TGF-β1 + P5 and TGF-β1 groups, we identified 353 upregulated genes and 1405 downregulated genes (Figure 4C). P5 treatment significantly reversed the gene expression changes induced by TGF-β1, particularly those related to ECM. GO enrichment analysis further confirmed that P5 regulates the expression of genes related to ECM organization, basement membrane, cell adhesion, and angiogenesis (Figure 4D), highlighting its role in inhibiting TGF-β1-induced fibrosis through these pathways. In summary, P5 exerts its anti-fibrotic effect by significantly reversing the gene expression changes induced by TGF-β1, especially those related to ECM and cell adhesion.

### 2.5. P5 Inhibits EMT Induced by TGF-β1 and BLM, Reducing Cell Migration and Mesenchymal Transition

**Figure 5:**
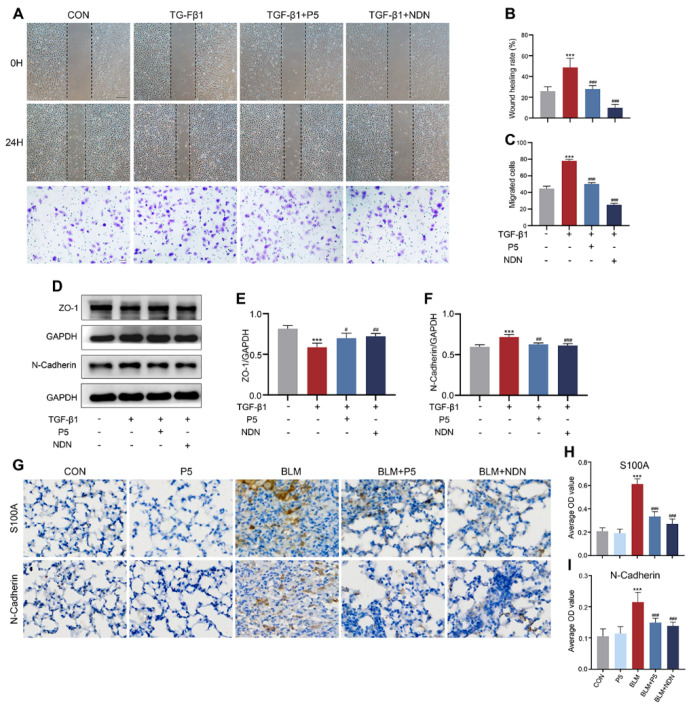
Inhibitory effect of P5 on TGF-β1-induced epithelial–mesenchymal transition (EMT). (**A**) Representative images of wound healing and Transwell migration assays in BEAS-2B cells. (**B**) Quantitative analysis of wound closure percentage. (**C**) Quantitative analysis of the number of migrated cells. (**D**) Western blot analysis of ZO-1 and N-Cadherin expression in BEAS-2B cells. (**E**) Quantification of ZO-1/GAPDH ratio. (**F**) Quantification of N-Cadherin/GAPDH ratio. (**G**) Representative images of immunohistochemical staining for S100A and N-Cadherin in lung tissue. (**H**) Quantitative analysis of the average optical density (OD) value of S100A. (**I**) Quantitative analysis of the average OD value of N-Cadherin. Data are presented as mean ± SD (*n* = 3–5). For comparisons among multiple groups, a one-way analysis of variance (ANOVA) was applied. *** *p* < 0.001 compared to the control group (CON); # *p* < 0.05, ## *p* < 0.01, ### *p* < 0.001 compared to the TGF-β1 group. The ‘+’ symbol represents ‘presence’ and the ‘–’ symbol represents ‘absence’. Scale bars = 200 µm in A, 100 µm in the below panels of A, and 15 µm in G.

To assess the inhibitory effect of P5 on TGF-β1-induced epithelial–mesenchymal transition (EMT), we evaluated its impact on cell migration and marker expression. Following TGF-β1 induction, P5 treatment for 24 h significantly inhibited the migration of BEAS-2B cells (Figure 5A–C). Western blot analysis showed that P5 treatment effectively restored epithelial marker ZO-1 and reduced mesenchymal marker N-Cadherin, reversing the changes induced by TGF-β1 (Figure 5D–F). Immunohistochemical staining of lung tissue further demonstrated that bleomycin (BLM) treatment significantly upregulated mesenchymal markers S100A and N-Cadherin, while P5 treatment reversed these effects, indicating inhibition of EMT (Figure 5G–I). In summary, P5 significantly reduces cell migration and mesenchymal transition by inhibiting EMT processes induced by TGF-β1 and BLM.

### 2.6. P5 Modulates TGF-β1-Induced Signaling Pathways and Gene Expression Changes

**Figure 6:**
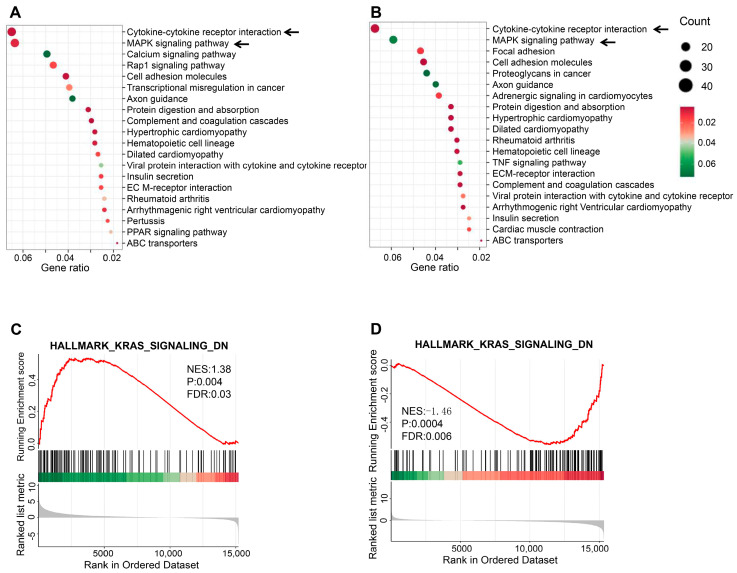
KEGG and GSEA analyses of the regulatory effect of P5 on TGF-β1-induced signaling pathways. (**A**): KEGG pathway enrichment analysis of differentially expressed genes between the TGF-β1-treated group and the control group (CON). The black arrows indicated the main pathways affected by TGF-β1. (**B**): KEGG pathway enrichment analysis of differentially expressed genes between the TGF-β1 + P5 group and the TGF-β1 group. The black arrows indicated the main pathways affected by P5. (**C**): GSEA analysis of the TGF-β1-treated group versus the control group (CON), showing significant enrichment of the upregulated KRAS signaling pathway. (**D**): GSEA analysis of the TGF-β1 + P5 group versus the TGF-β1 group, showing enrichment of the downregulated KRAS signaling pathway. NES: Normalized Enrichment Score; *p*-value and FDR value are used to assess enrichment significance. Arrows indicate pathways significantly enriched in each group.

To further investigate the regulatory effects of P5 on TGF-β1-induced signaling pathways, KEGG pathway analysis and gene set enrichment analysis (GSEA) were conducted on the differentially expressed genes (DEGs). KEGG pathway analysis revealed that TGF-β1 promotes fibrosis through pathways such as cytokine-cytokine receptor interactions and MAPK signaling (Figure 6A). P5 treatment significantly modulated these pathways, as evidenced by the enrichment analysis comparing the TGF-β1 + P5 and TGF-β1 groups (Figure 6B). GSEA further identified the KRAS signaling pathway as significantly activated during TGF-β1-induced fibrosis, while P5 treatment notably reduced its activity (Figure 6C,D). These findings demonstrate that P5 mitigates TGF-β1-induced fibrosis by regulating key pathways, including MAPK and KRAS signaling, and modulating cytokine-cytokine receptor interactions.

### 2.7. P5 Regulates Bleomycin-Induced Pulmonary Fibrosis Through the FGFR/MAPK Signaling Pathway

**Figure 7:**
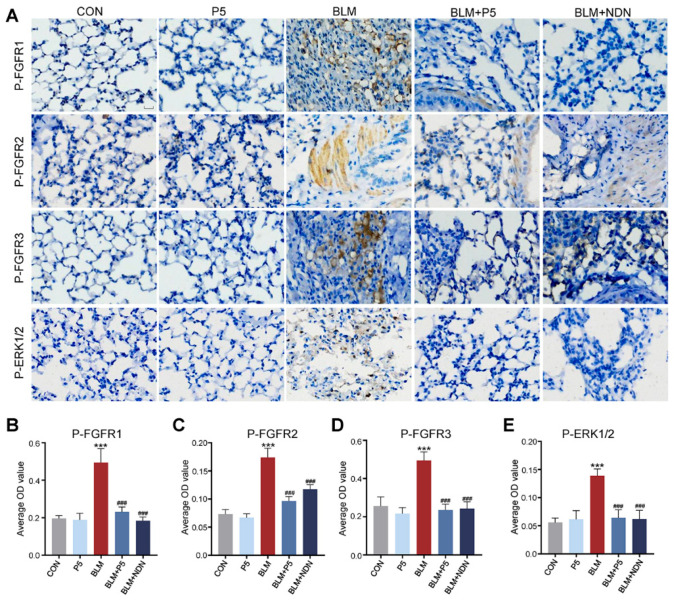
Inhibitory effect of P5 on bleomycin (BLM)-induced FGFR/MAPK signaling pathway. (**A**): Representative immunohistochemical images of P-FGFR1, P-FGFR2, P-FGFR3, and P-ERK1/2 in lung tissue. (**B**): Quantitative analysis of the average optical density (OD) value of P-FGFR1. (**C**): Quantitative analysis of the average OD value of P-FGFR2. (**D**): Quantitative analysis of the average OD value of P-FGFR3. (**E**): Quantitative analysis of the average OD value of P-ERK1/2. Data are presented as mean ± SD (*n* = 3). For comparisons among multiple groups, a one-way analysis of variance (ANOVA) was applied. *** *p* < 0.001 compared to the control group (CON); ### *p* < 0.001 compared to the BLM group. Scale bars = 15 µm in A.

Research has shown that high expression of FGF1 and its receptors (FGFRs) in the lungs of patients with idiopathic pulmonary fibrosis (IPF) activates the MAPK signaling pathway, promoting fibrosis [27]. To evaluate the effect of the derivative P5 on FGFR signaling pathways in bleomycin (BLM)-induced fibrosis, immunohistochemical analysis was performed on phosphorylated FGFR1, FGFR2, FGFR3, and ERK1/2 in lung tissues. BLM treatment significantly increased the levels of these phosphorylated proteins, indicating activation of the FGFR/MAPK signaling pathway and enhanced fibrosis. However, P5 treatment effectively reduced the expression of P-FGFR1, P-FGFR2, P-FGFR3, and P-ERK1/2, highlighting its anti-fibrotic effects through the inhibition of FGFR/MAPK pathway activation (Figure 7A–E). These results highlight the potential of P5 to mitigate fibrosis through suppression of FGFR/MAPK signaling.

## 3. Discussion

Idiopathic pulmonary fibrosis (IPF) is a chronic and progressive interstitial lung disease of unknown etiology, characterized by excessive fibroblast proliferation, extracellular matrix (ECM) deposition, and the gradual destruction of normal lung architecture, ultimately leading to irreversible lung function decline [39,40,41,42]. To replicate the pathological changes in human pulmonary fibrosis, the bleomycin (BLM)-induced pulmonary fibrosis model is widely used in preclinical research [43,44]. In this study, histological analyses, including HE and Masson staining, confirmed the successful establishment of the BLM-induced fibrosis model, showing typical alveolar inflammation, structural damage, and collagen deposition [45]. This work aimed to investigate the anti-fibrotic effects of the peptide P5 and elucidate its underlying mechanisms.

In the post-COVID-19 era, the prevalence of pulmonary fibrosis has significantly increased, underscoring the urgent need for safer and more effective treatments [41,46]. Current anti-fibrotic drugs, such as pirfenidone and Nintedanib, can slow lung function decline but are limited by significant side effects, including gastrointestinal disturbances and photosensitivity [47,48,49,50]. Peptide-based therapies have recently gained attention for their immunomodulatory, anti-inflammatory, and anti-fibrotic properties [51]. For instance, peptides like ToAP3 and Kefir peptide have demonstrated efficacy in reducing pulmonary fibrosis through mechanisms involving oxidative stress regulation and inflammation modulation [52,53]. Additionally, biomimetic lipid nanocomplexes with STAT3 inhibitory peptides have shown promise in targeting lung fibrosis [54]. Building on these findings, our study explored the therapeutic potential of P5, a small peptide derived from fibroblast growth factor 2 (FGF2), in treating pulmonary fibrosis. Previous studies demonstrated P5’s anti-inflammatory and anti-fibrotic effects in acne and cancer models, primarily through its inhibition of FGFR2 signaling [31,32]. Our findings extend these observations to pulmonary fibrosis, showing that P5 effectively reduces fibroblast activation and collagen deposition in bleomycin (BLM)-induced fibrosis model. Notably, P5 inhibited the expression of fibroblast activation markers, such as α-SMA, and attenuated extracellular matrix remodeling. These results highlight P5’s potential as a therapeutic agent targeting FGFR2-mediated fibrotic pathways.

Transforming growth factor-beta (TGF-β1), a key regulator of fibrosis, promotes fibroblast activation and epithelial–mesenchymal transition (EMT), both of which contribute to extracellular matrix deposition and fibrosis progression [55]. In our study, P5 inhibited TGF-β1-induced fibroblast activation and EMT, as evidenced by increased epithelial markers (e.g., ZO-1) and decreased mesenchymal markers (e.g., N-cadherin, α-SMA). These findings suggest that P5 alleviates fibrosis by restoring epithelial integrity and suppressing EMT, thus addressing critical drivers of IPF progression.

Transcriptomic analysis in our study revealed that the FGFR/MAPK signaling pathway plays a central role in the fibrosis process, with P5 primarily exerting its anti-fibrotic effects by modulating this pathway. While other pathways, such as Wnt/β-catenin, TGF-β/Smad, and JAK/STAT, are known to contribute to fibrosis initiation and progression [56,57,58]. Our findings suggest that P5 may indirectly interact with these pathways, potentially through synergistic effects. Recent work by Rob Guzy et al. [59] also emphasizes the critical role of FGF2/FGFR signaling in pulmonary fibrosis, demonstrating that central lung tissues in IPF show significant myofibroblast activation, a key driver of fibrosis progression. Consistent with these findings, our study suggests that P5, as an FGF2-derived peptide, alleviates fibrosis by inhibiting myofibroblast activity and fibroblast differentiation. In addition to modulating fibroblast activity, gene ontology (GO) enrichment analysis indicated that P5 may mitigate fibrosis by influencing angiogenesis. Given that fibrosis is frequently accompanied by abnormal blood vessel formation [60,61], P5’s ability to restore normal vascular structures could improve blood supply to fibrotic tissues, thereby alleviating fibrosis. Furthermore, P5’s potential regulation of neural signaling pathways, such as axon guidance and neuron projection, highlights unexplored mechanisms that warrant further investigation, especially in the context of pulmonary fibrosis [62]. Interestingly, P5 was also enriched in cardiac-related pathways, suggesting potential therapeutic applications beyond pulmonary fibrosis.

However, addressing challenges such as tissue-specific delivery, controlled release, and effective targeting will be essential for translating P5’s therapeutic potential into broader fibrosis treatments. Future studies should explore its synergistic effects with existing anti-fibrotic therapies, such as Nintedanib, and investigate its multi-pathway interactions using the bleomycin-induced pulmonary fibrosis mouse model, providing deeper mechanistic insights and supporting the development of comprehensive, multi-targeted therapeutic strategies. Peptides like P5 face clinical translation challenges, particularly in maintaining long-term stability and bioactivity. Although lyophilization and low-temperature storage are commonly used to preserve peptides, ensuring stability under clinical conditions remains a key concern [63]. Subcutaneous or intravenous injection is currently the most feasible delivery method, as oral administration is limited by enzymatic degradation in the gastrointestinal tract [64]. Advances in nanoparticle carriers or peptide conjugates could enhance P5’s bioavailability and stability, making it a more practical therapeutic option [65]. Additionally, further research is needed to evaluate P5’s pharmacokinetics, including its half-life, tissue distribution, and metabolic pathways, to ensure sustained therapeutic efficacy [66]. These factors will be critical in determining P5’s long-term feasibility as a treatment.

In conclusion, our study demonstrates that P5 effectively alleviates BLM-induced pulmonary fibrosis through two primary mechanisms: inhibiting epithelial–mesenchymal transition (EMT) and suppressing FGFR/MAPK signaling pathway activation. These actions collectively reduce fibroblast activation and collagen deposition, highlighting P5’s potential as a multi-target anti-fibrotic agent. While further investigations in other fibrosis models and preclinical studies are required, our findings provide a strong foundation for exploring P5’s clinical application as a novel therapeutic strategy for pulmonary fibrosis.

## 4. Materials and Methods

### 4.1. Reagents

P5 peptide was purchased from QYAOBI0 (ChinaPeptides Co., Ltd., Shanghai, China), TGF-β1 was obtained from Peprotech Company (Cranbury, NJ, USA), and BLM (bleomycin) was purchased from Selleck (Houston, TX, USA).

### 4.2. Experimental Animal Groups and Treatments

Male C57BL/6J mice (20–25 g) were obtained from the Institute of Laboratory Animal Science, Jinan University (Guangzhou, China). The mice were anesthetized with an intraperitoneal injection of 1.25% tribromoethanol solution (0.2 mL/10 g body weight). On the first day, 40 μL of bleomycin (BLM, 3.5 mg/kg) dissolved in pure water was administered via tracheal instillation to induce a pulmonary fibrosis model. Treatments were initiated on day 7 after confirming the successful establishment of the model. The experimental groups included a blank control group (tracheal instillation of an equivalent volume of saline), a model group (BLM), a model + P5 group (BLM + tail vein injection of P5, 100 mg/kg), and a positive control group (BLM + oral administration of Nintedanib, 60 mg/kg). Each group consisted of 6 mice. Mice in each group were weighed every other day starting from the model establishment. Two hours after the final administration, the mice were sacrificed, and lung tissue, along with other major organs, was collected for histopathological examination and biochemical analysis. All the processes involving animal experiments in this study were in accordance with the procedures of the Ethical Committee for Animal Experimentation (Note: approval number—LL-202212130005, and the ethical approval to perform animal experiments was from 13 December 2022), Jiangxi Zhonghong Boyuan Biotechnology Co., Ltd., Nanchang, China.

### 4.3. Micro-CT Imaging

The Quantum FX Micro CT (PerkinElmer, Inc., Waltham, MA, USA) was used to image the lungs of different groups of mice anesthetized with 1.5% isoflurane. Imaging parameters are set as follows: 90 kV tube voltage, 360° rotation, 30 frames per second, and a 6 min scan time, according to previous reports [67].

### 4.4. Assessment of Hydroxyproline Levels

The level of hydroxyproline (HYP) in the lung was measured using a commercial kit (A030–1–1, Nanjing Jiancheng Bioengineering Institute, Nanjing, China) with the manufacturer’s protocol. Lung tissue was accurately weighed; the pH was adjusted to neutral, and the sample volume was made up to 10 mL, followed by centrifugation to collect the supernatant. The supernatant (1 mL) was incubated with Reagent 1 for 10 min, then with Reagent 2 for 5 min, and finally with Reagent 3, followed by a 15-min incubation in a 60 °C water bath. The sample was then centrifuged, and the supernatant was measured at 550 nm for colorimetric analysis.

### 4.5. Serum Antioxidant Enzyme Activity Determination (MDA, SOD)

Serum was collected by centrifuging the blood samples at 3500 rpm (approximately 1640× *g*) for 15 min, and levels of MDA and SOD were measured according to the manufacturer’s instructions (Jiancheng, Huludao, China).

### 4.6. Cell Culture and Cell Viability Assay

BEAS-2B and MRC-5 cells were cultured in high-glucose DMEM supplemented with 10% FBS, while A549 cells were maintained in 1640 medium containing 10% FBS. After achieving adherence, the cells were switched to 0.5% low-serum medium for 12 h and then treated with 10 ng/mL TGF-β1 and/or varying concentrations of P5 (0–100 μM) for 24 h. Nintedanib (NDN, 1 μM) was used as a positive control. The cell viability was measured using a cell counting kit-8 (CCK8, ab228554, Abcam, Cambridge, UK) as previously described [68]. At least three independent experiments were performed.

### 4.7. Immunofluorescence (IF) Staining

Cells were seeded on coverslips and cultured to 50–70% confluence, followed by drug treatment for 24 h. After treatment, cells were fixed with 4% paraformaldehyde for 10–15 min and washed three times with PBS for 5 min each. After fixation, cells were permeabilized with 0.1–0.5% Triton X-100 at room temperature for 10–15 min, followed by three PBS washes. To reduce nonspecific binding, cells were blocked with 5–10% normal serum or 1% BSA at room temperature for 30–60 min. Primary antibodies were then applied and incubated overnight at 4 °C (details listed in Supplementary Information S1), followed by three PBS washes. Fluorescent secondary antibodies were added and incubated for 1 h at room temperature in the dark, followed by another three PBS washes. Nuclei were stained with DAPI or Hoechst for 5–10 min in the dark, followed by three PBS washes. After staining, coverslips were mounted with an anti-fade mounting medium. Images of the stained sections were captured using an epifluorescence microscope (Olympus IX51, Olympus Corporation, Tokyo, Japan; Leica DM 4000B, Leica Microsystems, Wetzlar, Germany).

### 4.8. Histology

The lungs were fixed in 4% paraformaldehyde for 24 h. The upper lobe of the left lung from each group was dehydrated using a graded alcohol series, cleared with xylene, embedded in paraffin, and sectioned into 5 μm slices. These sections were then stained with hematoxylin and eosin (H&E) and Masson’s trichrome. Six random areas from each lung section were photographed under a light microscope. Inflammatory lesions of H&E-stained lung tissue sections were scored using the classification system of Szapiel et al. [69]. Level 0: normal tissues without alveolitis; level 1: mild alveolitis with <20% lesions of the lung; level 2: moderate alveolitis with 20–50% lesions of the lung; level 3: severe alveolitis with >50% lesions of the lung [69]. The fibrotic areas in the lung tissue (stained blue with Masson’s trichrome) were quantified using Image-Pro Plus 6.0 software.

### 4.9. Immunohistochemical (IHC) Staining

Immunohistochemical (IHC) staining Lung tissue was fixed in 4% paraformaldehyde, dehydrated, embedded in paraffin, and sectioned at 4 µm thickness. Using the UltraSensitive Rapid Immunohistochemistry Kit (Elabscience^®^ Biotechnology Co., Ltd., Wuhan, China), sections were deparaffinized, rehydrated, and subjected to heat-induced antigen retrieval in citrate buffer. Endogenous peroxidase activity was blocked with hydrogen peroxide, followed by blocking with the kit’s blocking solution. Sections were incubated overnight at 4 °C with primary antibodies (details listed in Supplementary Information S1). Then washed three times with PBS (5 min each) and incubated in the dark at room temperature for 2.5 h with HRP-conjugated secondary antibodies (from the Elabscience^®^ kit). DAB staining was performed by treating sections with DAB substrate solution, observing for color development to the desired intensity, and then rinsing with tap water to stop the reaction. Sections were counterstained with hematoxylin to visualize cell nuclei. Finally, images of the stained sections were captured using a digital slide scanner (EasyScen, Motic, Xiamen, China).

### 4.10. Western Blot

Proteins from mouse lungs, BEAS-2B, MRC-5, or A549 cells were extracted using tissue homogenates in radioimmunoprecipitation assay (RIPA) buffer (Sigma, St. Louis, MO, USA) containing protease and phosphatase inhibitors. Protein concentrations were determined using a Bicinchoninic Acid (BCA) assay. The extracted proteins were separated by 10% SDS-PAGE and transferred to a polyvinylidene difluoride (PVDF) membrane (Millipore, MA, USA). The membrane was blocked with 5% non-fat milk and incubated with primary antibodies (details listed in Supplementary Information S1) in TBST buffer at 4 °C overnight with gentle shaking. Afterward, the membrane was incubated with secondary antibodies—either horseradish peroxidase (HRP)-conjugated goat anti-rabbit IgG (1:3000; Cell Signaling Technology, AB_2099233, Danvers, MA, USA) or HRP-conjugated goat anti-mouse IgG (1:3000; Cell Signaling Technology, AB_330924, MA, USA). The signals were developed using SuperSignal™ West Femto Chemiluminescent Substrate (Thermo Fisher, Rockford, IL, USA) and visualized with the Gel Doc™ XR+ System (Bio-Rad, version 3.1, Hercules, CA, USA). Chemiluminescent signals were captured and analyzed using ImageJ software (version 1.54m).

### 4.11. Colony Formation Assay

BEAS-2B and A549 cells were prepared as a single-cell suspension at a density of 600–2000 cells/well and seeded into 6-well plates, ensuring an equal number of cells per well. The cells were cultured at 37 °C with 5% CO_2_, and their growth was monitored daily. On the third day, the experimental group was treated with different drugs diluted in a complete medium, while the control group received an equal volume of the complete medium; the medium was replaced every 3 days. When single cells formed colonies of approximately 50 cells or when a visible difference was observed between groups, the culture was stopped, and the cells were washed 3–5 times with PBS. The cells were then fixed with 1 mL of 4% paraformaldehyde for 15–30 min, air-dried, stained with 1 mL of 0.2% crystal violet for 15–20 min, and washed with PBS until no background color remained. The plates were air-dried and photographed, and colonies were counted and analyzed.

### 4.12. Wound Healing Assay

BEAS-2B cells were seeded into 6-well plates and cultured in DMEM supplemented with 10% FBS. Once the cells reached confluence, a 10 μL pipette tip was used to create a scratch in the monolayer to induce a wound. The cells were then washed three times with sterile PBS to remove debris. BEAS-2B cells were incubated in serum-free DMEM under 5% CO_2_. The experimental groups included control (CON), TGF-β1, TGF-β1 + P5, and TGF-β1 + NDN. Images of the wound were captured at 0 h and 6 h post-scratch. For each treatment group, at least three wells were analyzed, and images were acquired using an inverted microscope (Nikon Eclipse Ti–U, Tokyo, Japan).

### 4.13. Transwell Migration Assay

In a 24-well plate, 300 μL of drug-containing medium with 20% FBS was added to each well (the lower chamber). A suspension of BEAS-2B cells was prepared at a concentration of 4 × 10^4^ cells/mL in a serum-free medium and mixed gently with the drug. The Transwell inserts (with an 8 μm pore size) were placed in the 24-well plate, and the cell suspension was carefully added to the upper chamber of each insert, ensuring no bubbles formed. The plate was incubated at 37 °C with 5% CO_2_ for approximately 24 h (the exact time may vary depending on cell migration speed and should be determined by a preliminary experiment). After incubation, the medium was removed, and the cells on the upper surface of the insert were gently wiped off with a cotton swab. Both the upper and lower chambers were fixed with 300 μL of 4% paraformaldehyde for 20–30 min at room temperature and then air-dried. The inserts were stained with 0.2% crystal violet for 20–30 min, and the stain was gently washed off with distilled water until the background was clear. The migrated cells were observed under a microscope, photographed, and counted. The experiments were conducted using a Nikon TI-FL microscope, and images were captured using a suitable camera. The acquired images were analyzed using the NIS-Elements software (version 3.22.09).

### 4.14. Molecular Docking

FGFR2 (ID: 1EV2) was downloaded from the Protein Data Bank (PDB) (https://www.rcsb.org/, 25 November 2023) for subsequent molecular docking experiments. The P5 peptide was derived by selecting the corresponding region from the FGF2 protein. Molecular docking between the P5 peptide and FGFR2 was performed using the docking server (https://gramm.compbio.ku.edu/gramm, 25 November 2023) with the PDB file format. For each docking simulation, 10 model predictions were generated, with model 1 selected as the optimal docking complex for further analysis. The docking results were then analyzed on the PDBe PISA website (https://www.ebi.ac.uk/msd-srv/prot_int/, 25 November 2023), which provides detailed information about the docking interactions. By inputting the PDB file of the docking complex, the number of hydrogen bonds, bond lengths, interacting amino acid residues, and the free energy associated with the docking process were evaluated.

### 4.15. RNA Sequencing (RNA-Seq) Transcript Profiling

BEAS-2B cells from the control group, TGF-β (10 ng/mL) treatment group, and TGF-β (10 ng/mL) combined with P5 (100 μM) treatment group (*n* = 3 per group) were harvested for RNA extraction. Total RNA was extracted using TRIzol reagent (Invitrogen, Carlsbad, CA, USA) according to the manufacturer’s instructions. Briefly, cells were lysed in TRIzol reagent, and the RNA was isolated by chloroform extraction and isopropanol precipitation. The quality and quantity of the RNA were assessed using a NanoDrop spectrophotometer (Thermo Fisher Scientific, Waltham, MA, USA) and agarose gel electrophoresis. RNA samples with an A260/A280 ratio between 1.8 and 2.0 were used for sequencing. The sequencing data were analyzed by GenePlus Bioinformatics Technology Co., Ltd. (Beijing, China) to identify differentially expressed genes (DEGs) among the experimental groups. The bioinformatics analysis included the generation of volcano plots to visualize DEGs, Gene Ontology (GO) analysis to identify enriched biological processes, molecular functions, and cellular components, KEGG pathway analysis to determine enriched pathways, and Gene Set Enrichment Analysis (GSEA) to explore significant gene set enrichments.

### 4.16. Data Analysis

Statistical analysis was implemented using the SPSS 20.0 statistical package program. Construction of statistical charts was carried out using the GraphPad Prism software package (GraphPad Software, version 9, San Diego, CA, USA). The data were presented as average ± SD. A comparison of the means between the two groups was performed using the Mann–Whitney U or Student’s *t*-test to estimate the differences. Multiple group comparisons of the means were performed by one-way analysis of variance (ANOVA). All animal experiment statistics data and the exact value of n are presented in the additional file: Supplementary results. *p* < 0.05 was deemed statistically significant.

## Data Availability

All data generated or analyzed during this study are included in the published article.

## Supplementary Materials

The following supporting information can be downloaded at: https://www.mdpi.com/article/10.3390/ijms26020517/s1.

## References

[1] King T.E., Pardo A., Selman M. (2011). Idiopathic pulmonary fibrosis. Lancet.

[2] Summer R., Chun P. (2024). Pressed for Understanding: Interstitial lung disease in Dry-Cleaning Workers. Am. J. Med Sci..

[3] Yatera K., Nishida C. (2024). Contemporary Concise Review 2023: Environmental and occupational lung diseases. Respirology.

[4] Poole J.A., Zamora-Sifuentes J.L., Vecillas L.D.L., Quirce S. (2024). Respiratory Diseases Associated with Organic Dust Exposure. J. Allergy Clin. Immunol. Pract..

[5] Sullivan D.I., Ascherman D.P. (2024). Rheumatoid Arthritis-Associated Interstitial Lung Disease (RA-ILD): Update on Prevalence, Risk Factors, Pathogenesis, and Therapy. Curr. Rheumatol. Rep..

[6] Obi O.N., Saketkoo L.A., Maier L.A., Baughman R.P. (2024). Developmental drugs for sarcoidosis. J. Autoimmun..

[7] Wang Y., Zhang J., Shao C. (2024). Cytological changes in radiation-induced lung injury. Life Sci..

[8] Borie R., Berteloot L., Kannengiesser C., Griese M., Cazes A., Crestani B., Hadchouel A., Debray M.P. (2024). Rare genetic interstitial lung diseases: A pictorial essay. Eur. Respir. Rev..

[9] Borie R., Ba I., Debray M.-P., Kannengiesser C., Crestani B. (2024). Syndromic genetic causes of pulmonary fibrosis. Curr. Opin. Pulm. Med..

[10] Giriyappagoudar M., Vastrad B., Horakeri R., Vastrad C. (2023). Study on Potential Differentially Expressed Genes in Idiopathic Pulmonary Fibrosis by Bioinformatics and Next-Generation Sequencing Data Analysis. Biomedicines.

[11] Koudstaal T., Wijsenbeek M.S. (2023). Idiopathic pulmonary fibrosis. Presse Med..

[12] Gupta R.S., Koteci A., Morgan A., George P.M., Quint J.K. (2023). Incidence and prevalence of interstitial lung diseases worldwide: A systematic literature review. BMJ Open Respir. Res..

[13] Podolanczuk A.J., Thomson C.C., Remy-Jardin M., Richeldi L., Martinez F.J., Kolb M., Raghu G. (2023). Idiopathic pulmonary fibrosis: State of the art for 2023. Eur. Respir. J..

[14] Ruaro B., Pozzan R., Confalonieri P., Tavano S., Hughes M., Matucci Cerinic M., Baratella E., Zanatta E., Lerda S., Geri P. (2022). Gastroesophageal Reflux Disease in Idiopathic Pulmonary Fibrosis: Viewer or Actor? To Treat or Not to Treat?. Pharmaceuticals.

[15] Chanda D., Otoupalova E., Smith S.R., Volckaert T., De Langhe S.P., Thannickal V.J. (2019). Developmental pathways in the pathogenesis of lung fibrosis. Mol. Asp. Med..

[16] Yang L., Zhou F., Zheng D., Wang D., Li X., Zhao C., Huang X. (2021). FGF/FGFR signaling: From lung development to respiratory diseases. Cytokine Growth Factor. Rev..

[17] Li L., Zhang S., Wei L., Wang Z., Ma W., Liu F., Qian Y. (2018). FGF2 and FGFR2 in patients with idiopathic pulmonary fibrosis and lung cancer. Oncol. Lett..

[18] Shimbori C., Bellaye P.-S., Xia J., Gauldie J., Ask K., Ramos C., Becerril C., Pardo A., Selman M., Kolb M. (2016). Fibroblast growth factor-1 attenuates TGF-β1-induced lung fibrosis. J. Pathol..

[19] Ramos C., Becerril C., Montaño M., García-De-Alba C., Ramírez R., Checa M., Pardo A., Selman M. (2010). FGF-1 reverts epithelial-mesenchymal transition induced by TGF-β1 through MAPK/ERK kinase pathway. Am. J. Physiol. Lung Cell. Mol. Physiol..

[20] Ramos C., Montaño M., Becerril C., Cisneros-Lira J., Barrera L., Ruíz V., Pardo A., Selman M. (2006). Acidic fibroblast growth factor decreases alpha-smooth muscle actin ex pression and induces apoptosis in human normal lung fibroblasts. Am. J. Physiol. Lung Cell. Mol. Physiol..

[21] Aguilar S., Scotton C.J., McNulty K., Nye E., Stamp G., Laurent G., Bonnet D., Janes S.M. (2009). Bone marrow stem cells expressing keratinocyte growth factor via an in ducible lentivirus protects against bleomycin-induced pulmonary fibrosis. PLoS ONE.

[22] MacKenzie B., Henneke I., Hezel S., Al Alam D., El Agha E., Chao C.-M., Quantius J., Wilhelm J., Jones M., Goth K. (2014). Attenuating endogenous Fgfr2b ligands during bleomycin-induced lung fi brosis does not compromise murine lung repair. Am. J. Physiol. Lung Cell. Mol. Physiol..

[23] Schütz K., Schmidt A., Schwerk N., Renz D.M., Gerard B., Schaefer E., Antal M.C., Peters S., Griese M., Rapp C.K. (2023). Variants in FGF10 cause early onset of severe childhood interstitial l ung disease: A detailed description of four affected children. Pediatr. Pulmonol..

[24] Lv Y.-Q., Cai G.-F., Zeng P.-P., Dhlamini Q., Chen L.-F., Chen J.-J., Lyu H.-D., Mossahebi-Mohammadi M., Ahmadvand N., Bellusci S. (2022). FGF10 Therapeutic Administration Promotes Mobilization of Injury-Activ ated Alveolar Progenitors in a Mouse Fibrosis Model. Cells.

[25] Joannes A., Brayer S., Besnard V., Marchal-Sommé J., Jaillet M., Mordant P., Mal H., Borie R., Crestani B., Mailleux A.A. (2015). FGF9 and FGF18 in idiopathic pulmonary fibrosis promote survival and m igration and inhibit myofibroblast differentiation of human lung fibro blasts in vitro. Am. J. Physiol. Lung Cell. Mol. Physiol..

[26] Zhang S., Yu D., Wang M., Huang T., Wu H., Zhang Y., Zhang T., Wang W., Yin J., Ren G. (2018). FGF21 attenuates pulmonary fibrogenesis through ameliorating oxidative stress in vivo and in vitro. Biomed. Pharmacother..

[27] MacKenzie B., Korfei M., Henneke I., Sibinska Z., Tian X., Hezel S., Dilai S., Wasnick R., Schneider B., Wilhelm J. (2015). Increased FGF1-FGFRc expression in idiopathic pulmonary fibrosis. Respir. Res..

[28] Landi C., Bergantini L., Cameli P., d’Alessandro M., Carleo A., Shaba E., Rottoli P., Bini L., Bargagli E. (2020). Idiopathic Pulmonary Fibrosis Serum proteomic analysis before and after nintedanib therapy. Sci. Rep..

[29] Easter M., Hirsch M.J., Harris E., Howze P.H.t., Matthews E.L., Jones L.I., Bollenbecker S., Vang S., Tyrrell D.J., Sanders Y.Y. (2024). FGF receptors mediate cellular senescence in the cystic fibrosis airwa y epithelium. JCI Insight.

[30] Yu Z.-H., Wang D.-D., Zhou Z.-Y., He S.-L., Chen A.-A., Wang J. (2012). Mutant soluble ectodomain of fibroblast growth factor receptor-2 IIIc attenuates bleomycin-induced pulmonary fibrosis in mice. Biol. Pharm. Bull..

[31] Su Z., Zhang Y., Cao J., Sun Y., Cai Y., Zhang B., He L., Zhang Z., Xie J., Meng Q. (2023). Hyaluronic acid-FGF2-derived peptide bioconjugates for suppression of FGFR2 and AR simultaneously as an acne antagonist. J. Nanobiotechnol..

[32] Zhang Y., Ouyang M., Wang H., Zhang B., Guang W., Liu R., Li X., Shih T.C., Li Z., Cao J. (2020). A cyclic peptide retards the proliferation of DU145 prostate cancer cells in vitro and in vivo through inhibition of FGFR2. MedComm.

[33] Katoh M., Loriot Y., Brandi G., Tavolari S., Wainberg Z.A., Katoh M. (2024). FGFR-targeted therapeutics: Clinical activity, mechanisms of resistanc e and new directions. Nat. Rev. Clin. Oncol..

[34] Kommalapati A., Tella S.H., Borad M., Javle M., Mahipal A. (2021). FGFR Inhibitors in Oncology: Insight on the Management of Toxicities i n Clinical Practice. Cancers.

[35] Roth G.J., Binder R., Colbatzky F., Dallinger C., Schlenker-Herceg R., Hilberg F., Wollin S.-L., Kaiser R. (2015). Nintedanib: From discovery to the clinic. J. Med. Chem..

[36] Roskoski R. (2020). The role of fibroblast growth factor receptor (FGFR) protein-tyrosine kinase inhibitors in the treatment of cancers including those of the u rinary bladder. Pharmacol. Res..

[37] Ong C.H., Tham C.L., Harith H.H., Firdaus N., Israf D.A. (2021). TGF-β-induced fibrosis: A review on the underlying mechanism and potential therapeutic strategies. Eur. J. Pharmacol..

[38] Chapman H.A. (2011). Epithelial-mesenchymal interactions in pulmonary fibrosis. Annu. Rev. Physiol..

[39] Richeldi L., Collard H.R., Jones M.G. (2017). Idiopathic pulmonary fibrosis. Lancet.

[40] Lederer D.J., Martinez F.J. (2018). Idiopathic Pulmonary Fibrosis. N. Engl. J. Med..

[41] Bonella F., Spagnolo P., Ryerson C. (2013). Current and Future Treatment Landscape for Idiopathic Pulmonary Fibros is. Drugs.

[42] Moss B.J., Ryter S.W., Rosas I.O. (2022). Pathogenic Mechanisms Underlying Idiopathic Pulmonary Fibrosis. Annu. Rev. Pathol..

[43] Ayilya B.L., Balde A., Ramya M., Benjakul S., Kim S.-K., Nazeer R.A. (2023). Insights on the mechanism of bleomycin to induce lung injury and assoc iated in vivo models: A review. Int. Immunopharmacol..

[44] Tashiro J., Rubio G.A., Limper A.H., Williams K., Elliot S.J., Ninou I., Aidinis V., Tzouvelekis A., Glassberg M.K. (2017). Exploring Animal Models That Resemble Idiopathic Pulmonary Fibrosis. Front. Med..

[45] Pei Z., Qin Y., Fu X., Yang F., Huo F., Liang X., Wang S., Cui H., Lin P., Zhou G. (2022). Inhibition of ferroptosis and iron accumulation alleviates pulmonary f ibrosis in a bleomycin model. Redox Biol..

[46] Somogyi V., Chaudhuri N., Torrisi S.E., Kahn N., Müller V., Kreuter M. (2019). The therapy of idiopathic pulmonary fibrosis: What is next?. Eur. Respir. Rev..

[47] Finnerty J.P., Ponnuswamy A., Dutta P., Abdelaziz A., Kamil H. (2021). Efficacy of antifibrotic drugs, nintedanib and pirfenidone, in treatme nt of progressive pulmonary fibrosis in both idiopathic pulmonary fibr osis (IPF) and non-IPF: A systematic review and meta-analysis. BMC Pulm. Med..

[48] Karampitsakos T., Juan-Guardela B.M., Tzouvelekis A., Herazo-Maya J.D. (2023). Precision medicine advances in idiopathic pulmonary fibrosis. EBioMedicine.

[49] Varone F., Sgalla G., Iovene B., Bruni T., Richeldi L. (2018). Nintedanib for the treatment of idiopathic pulmonary fibrosis. Expert Opin. Pharmacother..

[50] Takeda Y., Tsujino K., Kijima T., Kumanogoh A. (2014). Efficacy and safety of pirfenidone for idiopathic pulmonary fibrosis. Patient Prefer. Adherence.

[51] Li S., Li Y., Liu Y., Wu Y., Wang Q., Jin L., Zhang D. (2023). Therapeutic Peptides for Treatment of Lung Diseases: Infection, Fibros is, and Cancer. Int. J. Mol. Sci..

[52] Simon K.S., Coelho L.C., Veloso P.H.H., Melo-Silva C.A., Morais J.A.V., Longo J.P.F., Figueiredo F., Viana L., Silva Pereira I., Amado V.M. (2023). Innovative Pre-Clinical Data Using Peptides to Intervene in the Evolution of Pulmonary Fibrosis. Int. J. Mol. Sci..

[53] Lan Y.W., Chen Y.C., Yen C.C., Chen H.L., Tung M.C., Fan H.C., Chen C.M. (2024). Kefir peptides mitigate bleomycin-induced pulmonary fibrosis in mice through modulating oxidative stress, inflammation and gut microbiota. Biomed. Pharmacother..

[54] Keum H., Kim J., Yoo D., Kim T.W., Seo C., Kim D., Jon S. (2021). Biomimetic lipid Nanocomplexes incorporating STAT3-inhibiting peptides effectively infiltrate the lung barrier and ameliorate pulmonary fibrosis. J. Control. Release Off. J. Control. Release Soc..

[55] Frangogiannis N. (2020). Transforming growth factor-β in tissue fibrosis. J. Exp. Med..

[56] Li X., Liu X., Deng R., Gao S., Jiang Q., Liu R., Li H., Miao Y., Zhai Y., Zhang S. (2020). Betulinic acid attenuated bleomycin-induced pulmonary fibrosis by effe ctively intervening Wnt/β-catenin signaling. Phytomedicine.

[57] Boutanquoi P.-M., Burgy O., Beltramo G., Bellaye P.-S., Dondaine L., Marcion G., Pommerolle L., Vadel A., Spanjaard M., Demidov O. (2019). TRIM33 prevents pulmonary fibrosis by impairing TGF-β1 signalling. Eur. Respir. J..

[58] Yang F., Hou R., Liu X., Tian Y., Bai Y., Li J., Zhao P. (2022). Yangqing Chenfei formula attenuates silica-induced pulmonary fibrosis by suppressing activation of fibroblast via regulating PI3K/AKT, JAK/S TAT, and Wnt signaling pathway. Phytomedicine.

[59] Huang Y., Guzy R., Ma S.F., Bonham C.A., Jou J., Schulte J.J., Kim J.S., Barros A.J., Espindola M.S., Husain A.N. (2023). Central lung gene expression associates with myofibroblast features in idiopathic pulmonary fibrosis. BMJ Open Respir. Res..

[60] Amoakon J.P., Lee J., Liyanage P., Arora K., Karlstaedt A., Mylavarapu G., Amin R., Naren A.P. (2024). Defective CFTR modulates mechanosensitive channels TRPV4 and PIEZO1 and drives endothelial barrier failure. iScience.

[61] Fließer E., Jandl K., Lins T., Birnhuber A., Valzano F., Kolb D., Foris V., Heinemann A., Olschewski H., Evermann M. (2024). Lung Fibrosis Is Linked to Increased Endothelial Cell Activation and D ysfunctional Vascular Barrier Integrity. Am. J. Respir. Cell. Mol. Biol..

[62] Yegen C.H., Marchant D., Bernaudin J.F., Planes C., Boncoeur E., Voituron N. (2023). Chronic pulmonary fibrosis alters the functioning of the respiratory neural network. Front. Physiol..

[63] Maghsoudian S., Sajjadi E., Hadavi N., Soltani M., Karami Z., Abed Hamadi Al Qushawi A., Akrami M., Kalantari F. (2024). Biomedical applications of peptide-gold nanoarchitectonics. Int. J. Pharm..

[64] Chawathe A., Ahire V., Luthra K., Patil B., Garkhal K., Sharma N. (2025). Analytical and drug delivery strategies for short peptides: From manufacturing to market. Anal. Biochem..

[65] Martian P.C., Tertis M., Leonte D., Hadade N., Cristea C., Crisan O. (2025). Cyclic peptides: A powerful instrument for advancing biomedical nanotechnologies and drug development. J. Pharm. Biomed. Anal..

[66] Binder U., Skerra A. (2024). Strategies for extending the half-life of biotherapeutics: Successes and complications. Expert. Opin. Biol. Ther..

[67] Ruscitti F., Ravanetti F., Essers J., Ridwan Y., Belenkov S., Vos W., Ferreira F., KleinJan A., van Heijningen P., Van Holsbeke C. (2017). Longitudinal assessment of bleomycin-induced lung fibrosis by Micro-CT correlates with histological evaluation in mice. Multidiscip. Respir. Med..

[68] Wang M., Zhang P., Li Z., Yan Y., Cheng X., Wang G., Yang X. (2022). Different cellular mechanisms from low- and high-dose zinc oxide nanoparticles-induced heart tube malformation during embryogenesis. Nanotoxicology.

[69] Ashcroft T., Simpson J.M., Timbrell V. (1988). Simple method of estimating severity of pulmonary fibrosis on a numerical scale. J. Clin. Pathol..

