# Supplementary material for: An FGF2-Derived Short Peptide Attenuates Bleomycin-Induced Pulmonary Fibrosis by Inhibiting Collagen Deposition and Epithelial–Mesenchymal Transition via the FGFR/MAPK Signaling Pathway
Source: Int J Mol Sci. 2025 Jan 9;26(2):517. doi: 10.3390/ijms26020517 (PMC11764546; doi:10.3390/ijms26020517)
Supplement: Supplementary file 1 [file ijms-26-00517-s001.zip › Supplementary Figure.pdf]

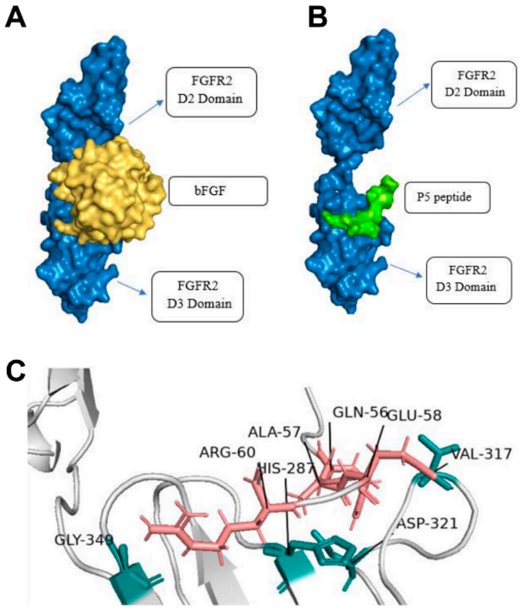

Supplementary Figure S1. Structural Analysis of P5 Binding to FGFR2.

A: Molecular model of the bFGF ligand (yellow) bound to the FGFR2 extracellular D2−D3 domains (blue). B: Molecular model showing the P5 (green) bound to the FGFR2 extracellular D2−D3 domains (blue). C: Detailed view of the binding interactions between the P5 and key residues of the FGFR2 D2−D3 domains, highlighting multiple non−covalent bonds.

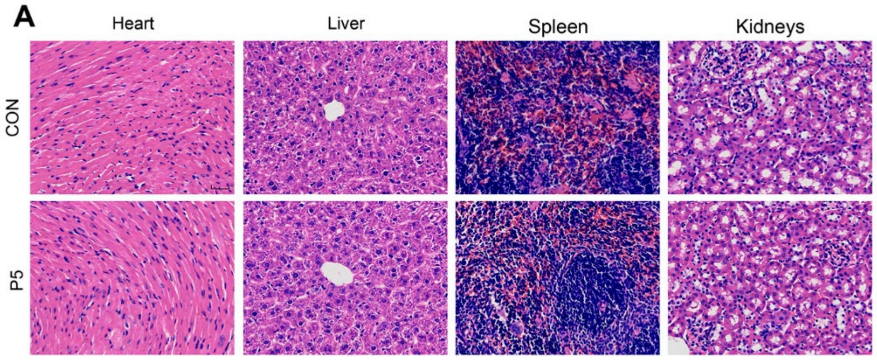

Supplementary Figure S2. Histological analysis of major organs

A: Representative images of hematoxylin and eosin (H&E) staining in the heart, liver, spleen, and kidneys from mice in the control (CON) group and P5−treated group. Scale bars =30 μm in A.

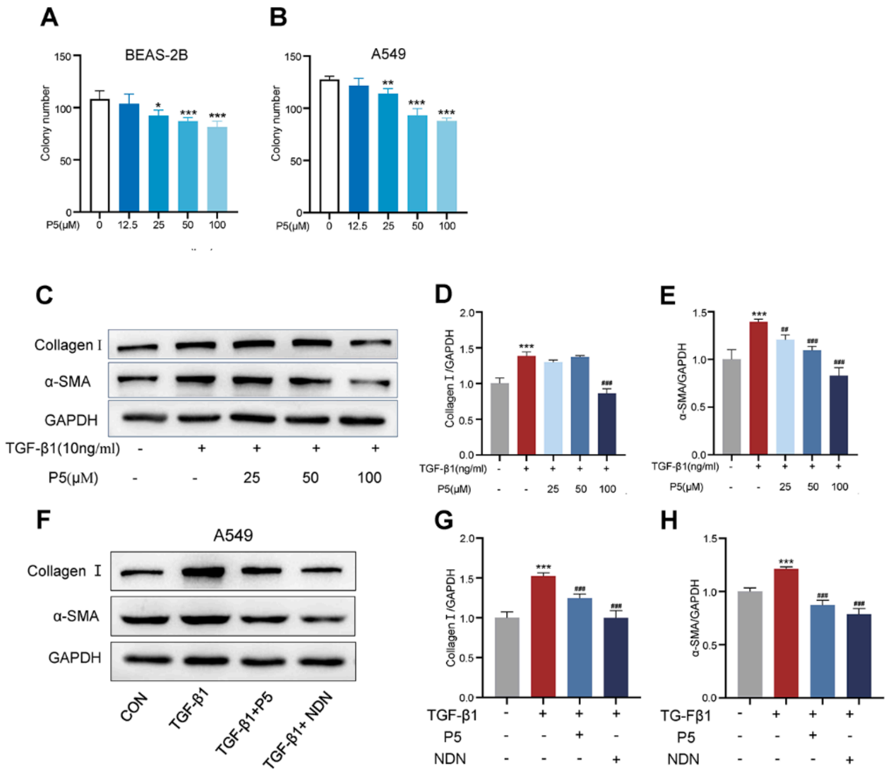

Supplementary Figure S3. Effect of P5 on TGF−β1−induced fibrosis marker expression

A−B: Quantification of colony formation in BEAS−2B and A549 cells. C: Western blot analysis showing the expression levels of Collagen I and α−SMA in BEAS−2B cells following TGF−β1 treatment with different concentrations of P5. D−E: Quantitative analysis of the Western blot results for BEAS−2B cells. F: Western blot analysis of α−SMA and Collagen I protein expression in A549 cells. G−H: Quantitative analysis of the Western blot results for A549 cells. Data are presented as mean ± SD (n = 3−4). *P < 0.05, **P < 0.01, ***P < 0.001 compared to the control group (CON); ###P < 0.001 compared to the TGF−β1 group. The '+' symbol represents 'presence' and the '–' symbol represents 'absence.
