# Supplementary material for: An FGF2-Derived Short Peptide Attenuates Bleomycin-Induced Pulmonary Fibrosis by Inhibiting Collagen Deposition and Epithelial–Mesenchymal Transition via the FGFR/MAPK Signaling Pathway
Source: Int J Mol Sci. 2025 Jan 9;26(2):517. doi: 10.3390/ijms26020517 (PMC11764546; doi:10.3390/ijms26020517)
Supplement: Supplementary file 1 [file ijms-26-00517-s001.zip › Supplementary information S1,S2.pdf]

Supplementary Information S1. Primary antibodies in this study

Primary antibody	Company	Catalog #	Dilutions
Collagen I	Cell signaling Technology	72026S	IF: 1:200<br>IHC: 1:200<br>WB: 1:1000
α-SMA	Cell signaling Technology	19245S	IF: 1:200<br>IHC: 1:200<br>WB: 1:1000
S100A	Proteintech	16105-1-AP	IHC: 1:100
N-Cadherin	BD Biosciences	610920	IHC: 1:200<br>WB: 1:2000
P-FGFR1	Thermo Fisher Scientific	44-1140G	IHC: 1:100
P-FGFR2	Thermo Fisher Scientific	PA5-105880	IHC: 1:100
P-FGFR3	Affinity	AF8439	IHC: 1:100
P-ERK1/2	Cell signaling Technology	4370S	IHC: 1:50
ZO-1	Proteintech	21773-1-AP	WB: 1:2000
GAPDH	Cell signaling Technology	97166S	WB: 1:5000

Supplementary Information S2. Statistical data (mean ± SD)

Fig1	Statistical index	CON	P5	BLM	BLM+P5	BLM+NDN	P
C	Right lung wet Weight（g）	0.088±0.003 N=4	0.085 ±0.003 N=4	0.103 ±0.009 N=4	0.093 ±0.004 N=4	0.090±0.007 N=4	CON & P5, P= 0.447; CON & BLM, P= 0.002; BLM & BLM+P5, P= 0.029; BLM & BLM+NDN, P= 0.005;
E	HYP content (μ g/mg)	1.068±0.051 N=6	1.072±0.042 N=6	1.231±0.026 N=6	1.082±0.039 N=6	1.086±0.026 N=6	CON & P5, P=0.863; CON & BLM, P<0.001; BLM & BLM+P5, P<0.001; BLM & BLM+NDN, P<0.001;
F	SOD(U/ml)	42.260±5.870 N=4	42.820±10.702 N=4	27.894±4.957 N=4	43.080±5.034 N=4	39.929±11.756 N=5	CON & P5, P=0.927; CON & BLM, P=0.029; BLM & BLM+P5, P=0.022; BLM & BLM+NDN, P=0.05;
G	MDA((μ mol/L)	7.283±0.987 N=5	5.570±1.119 N=4	13.225±4.683 N=6	5.707±1.294 N=4	6.342±1.748 N=3	CON & P5, P=0.366; CON & BLM, P=0.002; BLM & BLM+P5, P<0.001; BLM & BLM+NDN, P=0.003;

Fig2	Statistical index	CON	P5	BLM	BLM+P5	BLM+NDN	P
C	Ashcroft score (HE stain)	0.200±0.447 N=5	0.200±0.447 N=5	6.600±0.548 N=5	1.750±0.957 N=4	0.600±0.548 N=5	CON & P5, P=1.000; CON & BLM, P<0.001; BLM & BLM+P5, P<0.001; BLM & BLM+NDN, P<0.001;
D	Fibrotic percentage (%)	1.00±0.05 N=8	1.00±0.05 N=8	1.00±0.05 N=6	1.00±0.05 N=6	1.00±0.05 N=7	CON & P5, P=0.939; CON & BLM, P<0.001; BLM & BLM+P5, P<0.001; BLM & BLM+NDN, P<0.001;
E	Average OD value	0.296±0.013 N=3	0.294±0.016 N=3	0.380±0.007 N=3	0.330±0.009 N=3	0.310±0.018 N=3	CON & P5, P=0.831; CON & BLM, P<0.001; BLM & BLM+P5, P<0.001; BLM & BLM+NDN, P<0.001;
F	Average OD value	0.309±0.015 N=3	0.2710±0.040 N=3	0.405±0.006 N=3	0.334±0.010 N=3	0.321±0.028 N=3	CON & P5, P=0.076; CON & BLM, P<0.001; BLM & BLM+P5, P=0.004; BLM & BLM+NDN, P=0.001;

Fig3		Statistical index	CON	12.5	25	50	100	P
A	Beas-2b	Cell viability (% of control)	1.00±2.860 N=4	92.243±3.045 N=4	86.556±5.770 N=4	83.956±3.386 N=4	82.144±7.517 N=4	0 & 12.5, P=0.040; 0 & 25, P=0.001; 0 & 50, P<0.001; 0 & 100, P<0.001;
	A549	Cell viability (% of control)	1.00±2.580 N=3	83.010±3.350 N=4	80.746±4.472 N=4	78.870±8.305 N=4	74.134±5.377 N=4	0 & 12.5, P<0.001; 0 & 25, P<0.001; 0 & 50, P<0.001; 0 & 100, P<0.001;
	MRC-5	Cell viability (% of control)	1.00±6.219 N=4	94.030±4.383 N=4	92.427±4.228 N=4	85.841±6.4038 N=4	89.917±9.117 N=4	0 & 12.5, P=0.112; 0 & 25, P=0.049; 0 & 50, P=0.001; 0 & 100, P<0.001;

Fig3	Statistical index	CON	TGF-β1	TGF-β1+P5	TGF-β1+NDN	P
D	α-SMA Relative fluoresence intensity	1.00±0.060 N=4	1.334±0.068 N=4	1.010±0.139 N=4	1.044±0.083 N=4	CON & TGF-β1, P<0.001; TGF-β1 & TGF-β1+P5, P<0.001; TGF-β1 & TGF-β1+NDN, P<0.001;
E	Collagen I Relative fluoresence intensity	1.00±0.090 N=4	1.402±0.098 N=4	1.200±0.057 N=4	0.961±0.030 N=4	CON & TGF-β1, P<0.001; TGF-β1 & TGF-β1+P5, P=0.002; TGF-β1 & TGF-β1+NDN, P<0.001;
G	Collagen I/GAPDH	1.00±0.039 N=3	1.308±0.026 N=3	0.898±0.0421 N=3	0.720±0.088 N=3	CON & TGF-β1, P<0.001; TGF-β1 & TGF-β1+P5, P<0.001; TGF-β1 & TGF-β1+NDN, P<0.001;
H	α-SMA/GAPDH	1.00±0.026 N=3	1.309±0.086 N=3	0.924±0.029 N=3	0.807±0.063 N=3	CON & TGF-β1, P<0.001; TGF-β1 & TGF-β1+P5, P<0.001; TGF-β1 & TGF-β1+NDN, P<0.001;
J	α-SMA Relative fluoresence intensity	1.00±0.040 N=3	1.315±0.075 N=3	1.044±0.046 N=3	1.057±0.062 N=3	CON & TGF-β1, P<0.001; TGF-β1 & TGF-β1+P5, P<0.001; TGF-β1 & TGF-β1+NDN, P<0.001;
K	Collagen I Relative fluoresence intensity	1.00±0.017 N=3	1.250±0.109 N=3	1.041±0.100 N=3	0.971±0.036 N=3	CON & TGF-β1, P=0.044; TGF-β1 & TGF-β1+P5, P=0.010; TGF-β1 & TGF-β1+NDN, P=0.002;

M	Collagen I/GAPDH	1.00±0.132<br>N=3	1.590±0.050<br>N=3	1.181±0.069<br>N=3	0.990±0.058<br>N=3	CON & TGF-β1, P<0.001;<br>TGF-β1 & TGF-β1+P5, P<0.001;<br>TGF-β1 & TGF-β1+NDN, P<0.001;
N	α-SMA/GAPDH	1.00±0.063<br>N=3	1.37±0.039<br>N=3	1.14±0.018<br>N=3	0.893±0.066<br>N=3	CON & TGF-β1, P<0.001;<br>TGF-β1 & TGF-β1+P5, P<0.001;<br>TGF-β1 & TGF-β1+NDN, P<0.001;

Fig5	Statistical index	CON	TGF-β1	TGF-β1+P5	TGF-β1+NDN	P
B	Wound healing rate (%)	25.958±4.053<br>N=5	48.724±8.742<br>N=5	27.953±3.348<br>N=5	9.894±3.283<br>N=5	CON & TGF-β1, $P<0.001$;<br>TGF-β1 & TGF-β1+P5, $P<0.001$;<br>TGF-β1 & TGF-β1+NDN, $P<0.001$;
C	Migrated cells	44.667±2.887<br>N=3	78.000±1.732<br>N=3	50.000±1.732<br>N=3	25.000±2.000<br>N=3	CON & TGF-β1, $P<0.001$;<br>TGF-β1 & TGF-β1+P5, $P<0.001$;<br>TGF-β1 & TGF-β1+NDN, $P<0.001$;
E	ZO-1/GAPDH	0.816±0.038<br>N=3	0.587±0.051<br>N=3	0.699±0.061<br>N=3	0.723±0.033<br>N=3	CON & TGF-β1, $P<0.001$;<br>TGF-β1 & TGF-β1+P5, $P=0.019$;<br>TGF-β1 & TGF-β1+NDN, $P=0.008$;
F	N-Cadherin/GAPDH	0.598±0.025<br>N=3	0.717±0.033<br>N=3	0.626±0.184<br>N=3	0.614±0.053<br>N=3	CON & TGF-β1, $P<0.001$;<br>TGF-β1 & TGF-β1+P5, $P=0.002$;<br>TGF-β1 & TGF-β1+NDN, $P=0.001$;

Fig5	Statistical index	CON	P5	BLM	BLM+P5	BLM+NDN	P
H	Average OD value	0.208±0.030<br>N=3	0.192±0.034<br>N=3	0.613±0.044<br>N=3	0.334±0.042<br>N=3	0.271±0.042<br>N=3	CON & P5, P=0.627;<br>CON & BLM, P<0.001;<br>BLM & BLM+P5, P<0.001;<br>BLM & BLM+NDN, P<0.001;
I	Average OD value	0.105±0.024<br>N=3	0.114±0.022<br>N=3	0.214±0.032<br>N=3	0.149±0.014<br>N=3	0.139±0.012<br>N=3	CON & P5, P=0.629;<br>CON & BLM, P<0.001;<br>BLM & BLM+P5, P=004;<br>BLM & BLM+NDN, P=0.002;

Fig7	Statistical index	CON	P5	BLM	BLM+P5	BLM+NDN	P
B	Average OD value	0.196±0.014<br>N=3	0.188±0.036<br>N=3	0.494±0.074<br>N=3	0.230±0.026<br>N=3	0.184±0.020<br>N=3	CON & P5, P=0.805;<br>CON & BLM, P<0.001;<br>BLM & BLM+P5, P<0.001;<br>BLM & BLM+NDN, P<0.001;
C	Average OD value	0.073±0.008<br>N=3	0.067±0.007<br>N=3	0.174±0.016<br>N=3	0.096±0.008<br>N=3	0.117±0.008<br>N=3	CON & P5, P=0.468;<br>CON & BLM, P<0.001;<br>BLM & BLM+P5, P<0.001;<br>BLM & BLM+NDN, P<0.001;
D	Average OD value	0.255±0.048<br>N=3	0.216±0.032<br>N=3	0.494±0.045<br>N=3	0.235±0.031<br>N=3	0.242±0.036<br>N=3	CON & P5, P=0.243;<br>CON & BLM, P<0.001;<br>BLM & BLM+P5, P<0.001;<br>BLM & BLM+NDN, P<0.001;
E	Average OD value	0.056±0.008<br>N=3	0.061±0.016<br>N=3	0.139±0.012<br>N=3	0.064±0.014<br>N=3	0.062±0.016<br>N=3	CON & P5, P=0.628;<br>CON & BLM, P<0.001;<br>BLM & BLM+P5, P<0.001;<br>BLM & BLM+NDN, P<0.001;

Fig. S3	Statistical index	CON	12.5	25	50	100	P
A	Colony number	108.33±7.638<br>N=3	103.667±9.292<br>N=3	92.333±5.132<br>N=3	87.000±3.464<br>N=3	81.333±5.686<br>N=3	0 & 12.5, P=0.404;<br>0 & 25, P=0.014;<br>0 & 50, P=0.003;<br>0 & 100, P<0.001;
B	Colony number	127.500±3.109<br>N=4	121.500±7.000<br>N=4	114.000±4.761<br>N=4	93.000±6.683<br>N=4	87.750±2.872<br>N=4	0 & 12.5, P=0.122;<br>0 & 25, P=0.002;<br>0 & 50, P<0.001;<br>0 & 100, P<0.001;

Fig. S3	Statistical index	CON	TGF-β1	TGF-β1+25	TGF-β1+50	TGF-β1+100	P
D	Collagen I/GAPDH	1.00±0.079 N=3	1.387±0.056 N=3	1.299±0.030 N=3	1.369±0.021 N=3	0.860±0.069 N=3	CON & TGF-β1, P<0.001; TGF-β1 & TGF-β1+25, P=0.081; TGF-β1 & TGF-β1+50, P=0.696; TGF-β1 & TGF-β1+100, P<0.001;
E	α-SMA/GAPDH	1.00±0.101 N=3	1.395±0.028 N=3	1.206±0.050 N=3	1.094±0.043 N=3	0.829±0.085 N=3	CON & TGF-β1, P<0.001; TGF-β1 & TGF-β1+25, P=0.006; TGF-β1 & TGF-β1+50,P<0.001; TGF-β1 & TGF-β1+100,P<0.001;

Fig. S3	Statistical index	CON	TGF-β1	TGF-β1+P5	TGF-β1+NDN	P
G	Collagen I/GAPDH	1.00±0.074<br>N=3	1.522±0.041<br>N=3	1.248±0.049<br>N=3	0.998±0.091<br>N=3	CON & TGF-β1, P<0.001;<br>TGF-β1 & TGF-β1+P5, P<0.001;<br>TGF-β1 & TGF-β1+NDN, P<0.001
H	α-SMA/GAPDH	1.00±0.035<br>N=3	1.213±0.019<br>N=3	0.872±0.046<br>N=3	0.787±0.052<br>N=3	CON & TGF-β1, P<0.001;<br>TGF-β1 & TGF-β1+P5, P<0.001;<br>TGF-β1 & TGF-β1+NDN, P<0.001
